# Urinary Morbidity in Men Treated With Stereotactic Body Radiation Therapy (SBRT) for Localized Prostate Cancer Following Transurethral Resection of the Prostate (TURP)

**DOI:** 10.3389/fonc.2020.00555

**Published:** 2020-05-05

**Authors:** Abigail Pepin, Nima Aghdam, Sarthak Shah, Shaan Kataria, Harry Tsou, Subhradeep Datta, Malika Danner, Marilyn Ayoob, Thomas Yung, Siyuan Lei, Marie Gurka, Brian T. Collins, Pranay Krishnan, Simeng Suy, Ryan Hankins, John H. Lynch, Sean P. Collins

**Affiliations:** ^1^School of Medicine and Health Sciences, George Washington University, Washington, DC, United States; ^2^Department of Radiation Medicine, Georgetown University Hospital, Washington, DC, United States; ^3^Columbian College of Arts and Sciences, George Washington University, Washington, DC, United States; ^4^Department of Radiology, Georgetown University Hospital, Washington, DC, United States; ^5^Department of Urology, Georgetown University Hospital, Washington, DC, United States

**Keywords:** prostate cancer, SBRT, CyberKnife, benign prostatic hyperplasia, IPSS, EPIC-26, quality of life, common toxicity criteria (CTC)

## Abstract

**Background:** Clinical data suggest that stereotactic body radiation therapy (SBRT) provides similar clinical outcomes as other radiation modalities for prostate cancer. However, data reporting on the safety of SBRT after TURP is limited. Herein, we report our experience using SBRT to deliver hypofractionated radiotherapy in patients with a history of TURP including physician-reported toxicities and patient-reported quality of life.

**Methods:** Forty-seven patients treated with SBRT from 2007 to 2016 at Georgetown University Hospital for localized prostate carcinoma with a history of prior TURP were included in this retrospective analysis. Treatment was delivered using the CyberKnife® (Accuray Incorporated, Sunnyvale, CA) with doses of 35 Gy or 36.25 Gy in 5 fractions without prostatic urethral sparing. Toxicities were recorded and scored using the CTCAE v.4. Cystoscopy findings were retrospectively reviewed. Urinary quality of life data was assessed using the International Prostate Symptom Scoring (IPSS) and Expanded Prostate Cancer Index Composite 26 (EPIC-26). A Wilcoxon signed-rank sum test was used to determine if there was a statistically significant increase or decrease in IPSS or EPIC scores between timepoints. Minimally important differences were calculated by obtaining half the standard deviation at time of start of treatment.

**Results:** Forty-seven patients at a median age of 72 years (range 63–84) received SBRT. The mean follow-up was 4.7 years (range 2–10 years). Late Grade 2 and grade 3 urinary toxicity occurred in 23 (48.9%) and 3 (6.4%) men, respectively. There were no Grade 4 or 5 toxicities. Approximately 51% of patients experienced hematuria following treatment. Mean time to hematuria was 10.5 months. Twenty-five cystoscopies were performed during follow-up and the most common finding was hyperemia, varices of the bladder neck/TURP defect, and/or necrotic tissue in the TURP defect. Baseline urinary QOL composite scores were low, but they did not clinically significantly decline in the first 2 years following treatment.

**Conclusions:** In patients with prior TURP, prostate SBRT was well-tolerated. GU toxicity rates were comparable to similar patients treated with conventionally fractionated radiation therapy. Urinary quality of life was poor at baseline, but did not worsen clinically over time. Stricter dosimetric criteria could potentially improve the rate of high-grade late toxicity, but may increase the risk of peri-urethral recurrence.

## Background

While conventionally fractionated and moderately hypofractionated intensity modulated radiation therapy (IMRT) are the most commonly employed modalities for clinically localized prostate cancer (1), the utilization of ultra-hypofractionated treatment is increasing (2). Stereotactic Body Radiation Therapy (SBRT) with its increased accuracy, intrafraction image guidance, and reduced treatment margins may allow for a more convenient and effective form of external radiation therapy (3, 4). Emerging data from randomized trials suggest that this approach may provide similar clinical outcomes as other radiation modalities with high rates of biochemical control and low rates of grade 3 and higher toxicities (5, 6). The Hypo-RT-PC randomized phase III trial has shown identical biochemical disease-free survival and similar toxicities comparing standard fractionation with ultra-hypofractionated SBRT (5). Based on these reports, as well as patient preference for an abbreviated course of treatment, SBRT utilization is likely to continue to increase.

Benign prostatic hyperplasia (BPH) is a common problem in elderly men with >50% of men over the age of 50 experiencing it (7). Alpha_1_-receptor antagonists with or without 5-alpha-reductase blockers are used as first line therapy for symptomatic relief in patients with moderate to severe BPH (8). Transurethral resection of the prostate (TURP) is the standard of care for medical refractory BPH (8). While TURP relieves bothersome urinary obstruction, urinary function in general does not improve to baseline due to residual bladder injury. Post-TURP related complications include incontinence (in up to 11%), urethral strictures (in up to 10%), and bladder neck contractures (in up to 9%) (9). Unfortunately, BPH can regrow into the TURP defect, causing a recurrence of obstructive and irritative symptoms and the need for a repeat TURPs (3–15%) (9).

External beam radiation therapy (EBRT) causes predictable urinary side effects. Radiation urethitis/cystitis occurs months to years after radiation therapy of localized prostate cancer (10). Patients with radiation-induced cystitis experience hematuria, dysuria, frequency/urgency, incontinence, and retention (11). Retention and/or dysuria are commonly managed with alpha_1_-receptor antagonists (12, 13) while urinary, frequency/urgency and resulting incontinence are managed with anti-muscarinics (14). Cystoscopic evaluation for hematuria commonly reveals hyperemia, congested mucosa, and neovascularization (15). Prostate-related hematuria is commonly treated by 5a-reductase inhibitors (16). The risk of urethritis/cystitis is dependent upon both the total radiation dose and the volume of the urethra/bladder in the high dose area (17). Patient characteristics, such as a history of prior urethral procedures and/or chronic anticoagulation therapy (18) may increase an individual patient's risk of clinically significant urethritis/cystitis.

Data regarding the safety of SBRT after TURP is limited to highly selected patients (19, 20). The aim of our study is to report on radiation-related toxicities using common terminology for common adverse events (CTCAE v4) in unselected patients who have a history of TURP and have undergone SBRT for treatment of their prostate cancer. We also assessed baseline quality of life of post-TURP patients and the impact of subsequent SBRT treatment using validated instruments (EPIC-26 and IPSS). We report on quality of life measures in urinary incontinence and irritative/obstructive domains.

## Methods

### Patient Selection

**Figure 1:**
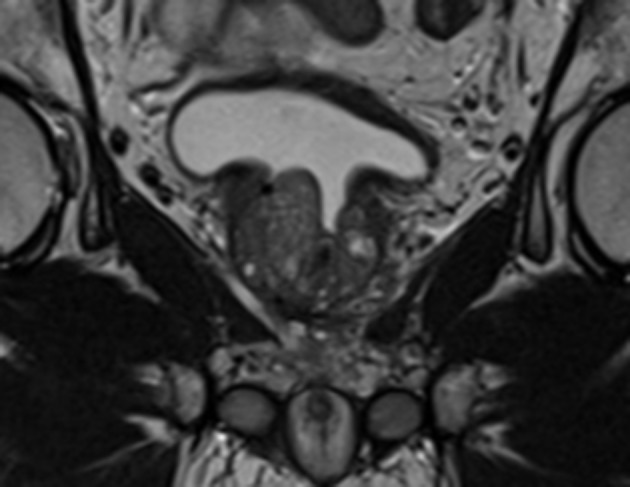
Coronal T2-weighted MRI revealing visible TURP defect.

The Georgetown University Institutional Review Board (IRB) approved this single institution retrospective review of prospectively collected toxicity and quality of life (QoL) data (IRB#: 2009-510). All individuals diagnosed with localized prostate cancer who received SBRT at MedStar Georgetown University Hospital from 2007 to 2016 were eligible for inclusion. In order to be included in this study, patients were required to have at least one TURP procedure prior to SBRT with a minimum of 24 months follow up following radiation treatment. Patients who had undergone minimally invasive procedures such as transurethral needle ablation or laser vaporization techniques were excluded from the current report. Figure 1 demonstrates a sagittal section of a T2 weighted MRI revealing visible TURP defect.

### SBRT Treatment Planning and Delivery

Simulation, contouring, and treatment planning were conducted based on an institutional protocol (21). Patients underwent a treatment planning CT and pelvic MRI at least 1 week after placement of 4–6 gold fiducial markers in the prostate. The clinical target volume (CTV) included the prostate and the proximal seminal vesicles. The PTV equaled the CTV expanded 3 mm posteriorly and 5 mm in all other dimensions. The prescription dose was 35-36.25 Gy to the PTV delivered in five fractions of 7–7.25 Gy over 1–2 weeks. The bladder and membranous urethra were contoured structures and evaluated with dose-volume histogram analysis during treatment planning using Multiplan (Accuray Inc., Sunnyvale, CA) inverse treatment planning. The empty bladder volume receiving 37 Gy was limited to <5 cc. The membranous urethra dose-volume histogram (DVH) goal was <50% the volume receiving 50% of the prescribed dose. The prescription isodose line was limited to >75% which limited the maximum prostatic urethra dose to 133% of the prescription dose.

### Follow-Up and Statistical Analysis

Assessment of toxicity was performed on the first day of treatment and during subsequent follow-up visits. The utilization of alpha_1_-receptor antagonists, anti-muscarinics, 5-alpha-reductase blockers, and anti-coagulants was documented at each visit. Toxicities at each time point were scored using the CTCAE v4. We defined acute toxicity as toxicity occurring in under 3 months post-treatment; all other toxicities after 3 months were considered late toxicity. Both transient and chronic toxicities were included and are reported here. Genitourinary toxicities included hematuria, dysuria, incontinence, urinary urgency/frequency, and urinary retention (21). Grade 1 toxicities included those who reported side effects not requiring medication for management. Grade 2 toxicities included requirement to start a new medication, increase the dose of medication, or if pre-treatment Grade 1 toxicities increased in severity. Grade 3 toxicities included those which required surgical intervention including repeat TURP. The highest GU toxicity was determined across time points and reported here.

Cross-sectional assessment of the quality of life using the IPSS and EPIC-26 questionnaires were assessed on the first day of treatment and during subsequent follow-ups at 1-month post-treatment, every 3 months during the first-year post-treatment, every 6 months after the second year, then yearly. The score for each EPIC-26 domain was calculated as a weighted percentage based on urinary irritative/obstructive and incontinence domains. Domain scores were obtained by calculating an average across the time point. A Wilcoxon signed-rank sum test was used to determine if there was any significant increase or decrease in EPIC score domains and combined IPSS score at different timepoints. A chi-square test was used to compare pad usage over time. Minimally important differences were calculated by obtaining half the standard deviation at time of start of treatment. Prism v8.3 (GraphPad Software, San Diego, CA) was used for statistically analysis. *P* < 0.05 was considered statistically significant.

## Results

Forty-seven prostate cancer patients with at least one prior TURP were treated per our institutional SBRT protocol from 2007 to 2016. The mean follow-up was 4.7 years (range 2–10 years). Table 1 provides patient characteristics. They were ethnically diverse with a median age of 72 years (range 63–84 years). Twelve patients were low-risk, 28 patients were intermediate-risk, and seven patients were high risk per the D'Amico Risk Classification (22). Seventeen patients also received androgen deprivation therapy (ADT). The median prostate volume was 42 cc (11.5–140 cc). The TURP procedure occurred months to years prior to SBRT with six patients treated with multiple prior TURPs. Comorbidities were common in our population (average Charlson Comorbidity Index (CCI) = 1.7, CCI >2 61.7%). Chronic anticoagulant use was noted in 40.4% of patients. A roughly equal percentage were treated with 35 Gy and 36.25 Gy in five fractions (23).

**Table 1:** Patient characteristics and treatment.

	**Percent of Patients (*n* = 47)**
**Age (years): Median 72 (63–84)**	
60–69 70–79 >80	36.2% (17) 48.9% (23) 14.9% (7)
**Race**	
White Black Other	48.9% (23) 38.3% (18) 12.8% (6)
**Time from TURP to SBRT**	
<12 mon 1–5 yrs 5–10 yrs >10 years Unknown	14 12 6 7 8
**# of TURP procedures**	
1 2 >3	87.2% (41) 10.6% (5) 2.1% (1)
**Prostate volume (cc)**	Median 42 (11.6–140)
**Pre-treatment PSA (ng/ml): Median 6.5 (1.8–17)**	
<10 >10 and <20	78.7% (37) 21.3% (10)
**T stage**	
T1b–T2a T2b–T2c	80.9% (38) 19.1% (9)
**Gleason score**	
6 7 8–9	29.8% (14) 55.3% (26) 14.9% (7)
**Charlson comorbidity Index**	
0 1 >2	27.7% (13) 10.6% (5) 61.7% (29)
**Risk group (D'Amico)**	
Low Intermediate High	25.5% (12) 59.6% (28) 14.9% (7)
**Hormone therapy**	
Yes No	36.2% (17) 63.8% (30)
**Anticoagulation**	
Anticoagulation No anticoagulation	40.4% (19) 59.6% (28)
**SBRT dose**	
35 36.25	57.4% (27) 42.6% (20)

**Figure 2:**
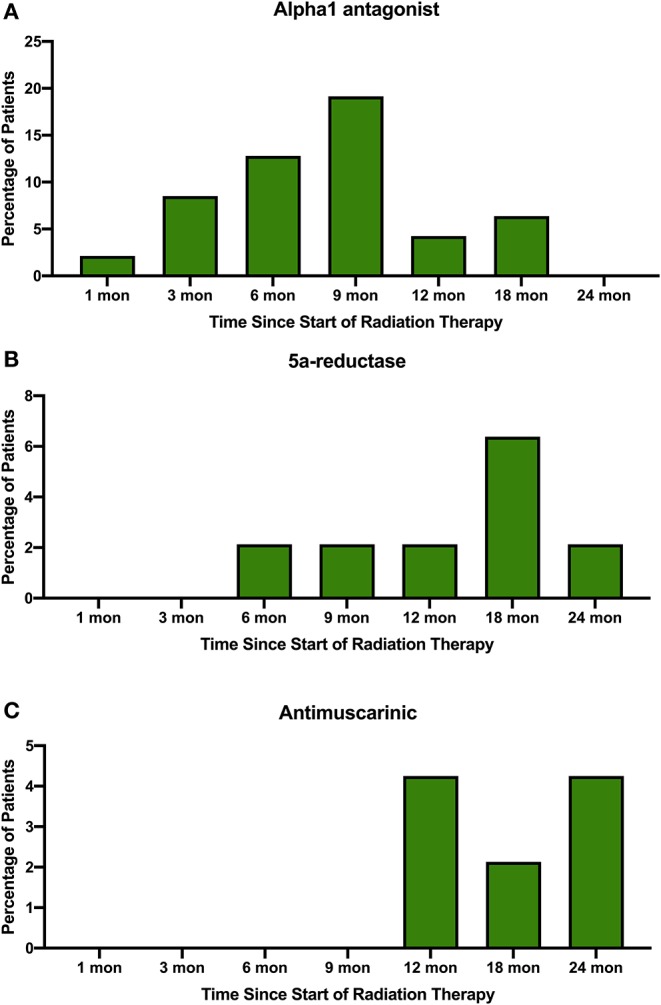
Percentage of patients prescribed **(A)** alpha_1_ antagonists, **(B)** 5a-reductase inhibitors, and **(C)** antimuscarinic agents to treat symptoms in the months following SBRT for their prostate cancer.

Acute and late urinary toxicities are reported in Table 2. Low grade acute toxicities were common. Utilization of alpha_1_ antagonists, 5a-reductase inhibitors, and antimuscarinic agents for symptomatic management over time are shown in Figure 2. One patient with an enlarged prostate (85 cc) and two prior TURPs experienced an acute Grade 3 hematuria requiring cauterization. Late Grade 2 and grade 3 urinary toxicity occurred in 23 (48.9%) and 3 (6.4%) men, respectively. Fifty-one percent of patients experienced hematuria. The mean time to hematuria was 10.5 months. Late Grade 2 hematuria requiring 5a-reductase inhibitors was observed in 12.8% of patients (Table 2). Twenty-five patients underwent cystoscopy to evaluate the etiology of the bleeding (Table 3). The most common findings were a hyperemic bladder neck/TURP defect with or without neovascularization and/or necrotic tissue. One patient required fulguration of varices and two patients required resection of necrotic tissue (Grade 3 toxicity) to stop the bleeding. Hematuria resolved prior to the next follow-up in 82% of patients. The newly diagnosed stricture rate was 4.3% in our population.

**Table 2:** Summary of CTC graded acute (defined as toxicity under 3 months) and late genitourinary (GU) toxicities (defined as toxicity over 3 months).

	**None**	**Grade 1**	**Grade 2**	**Grade 3**
**ACUTE**				
Hematuria^*^	40	6	0	1
Dysuria	27	20	0	0
Frequency/Urgency	11	36	0	0
Incontinence	25	22	0	0
Retention	18	23	6	0
**Overall**	**6 (12.7%)**	**34 (72.3%)**	**6 (12.8%)**	**1 (2.1%)**
**LATE**				
Hematuria^*^	25	13	6	3
Dysuria	24	20	3	0
Frequency/Urgency	3	38	6	0
Incontinence	16	24	7	0
Retention	9	25	11	2
**Overall**	**1 (2.1%)**	**20 (42.5%)**	**23 (48.9%)**	**3 (6.4%)**

* *There was a resolution of hematuria in 82% of patients by the next time point*.

**Table 3:** Cystoscopy results.

**Patient**	**Age**	**CCI**	**# of TURPs**	**Anticoagulant use**	**Cystoscopy findings**
1	64	0	2	None	Normal
2	72	0	2	None	TURP defect varices
3	67	2	1	None	Hyperemic median lobe
4	82	0	1	Yes	Enlarged prostate
5	66	5	1	Yes	Normal
6	74	2	1	None	Necrotic tissue in TURP defect
7	80	3	1	None	Normal
8	74	3	1	Yes	TURP defect varices
9	65	2	1	None	TURP defect varices
10	80	0	2	None	Normal
11	75	0	1	None	Normal
12	74	2	1	Yes	Hyperemic prostatic urethra; Bulbar stricture
13	66	2	1	None	Enlarged prostate
14	70	2	1	Yes	TURP defect varices
15	84	1	2	Yes	Normal
16	69	2	1	Yes	Bulbar stricture
17	63	2	1	Yes	TURP defect varices
18	70	3	1	None	Hyperemia of TURP defect
19	69	5	1	None	Bladder neck contracture
20	67	1	2	None	Necrotic tissue in TURP defect
21	72	2	1	Yes	Normal
22	65	2	Multiple	None	TURP defect varices
23	71	4	1	Yes	Enlarged prostate
24	84	2	1	Yes	Penile urethral stricture
25	76	0	1	Yes	Normal

**Figure 3:**
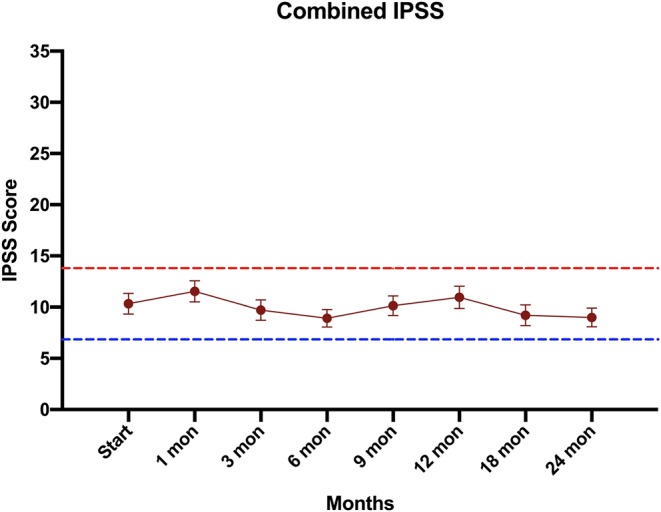
Urinary quality of life using the International Prostate Symptom Scoring (IPSS) score. The graphs show unadjusted changes in average scores over time for each domain. IPSS scores range from 0 – 35 with higher values representing worsening urinary symptoms. Error bars indicate SEM. The dashed lines represent the minimally important difference values.

**Figure 4:**
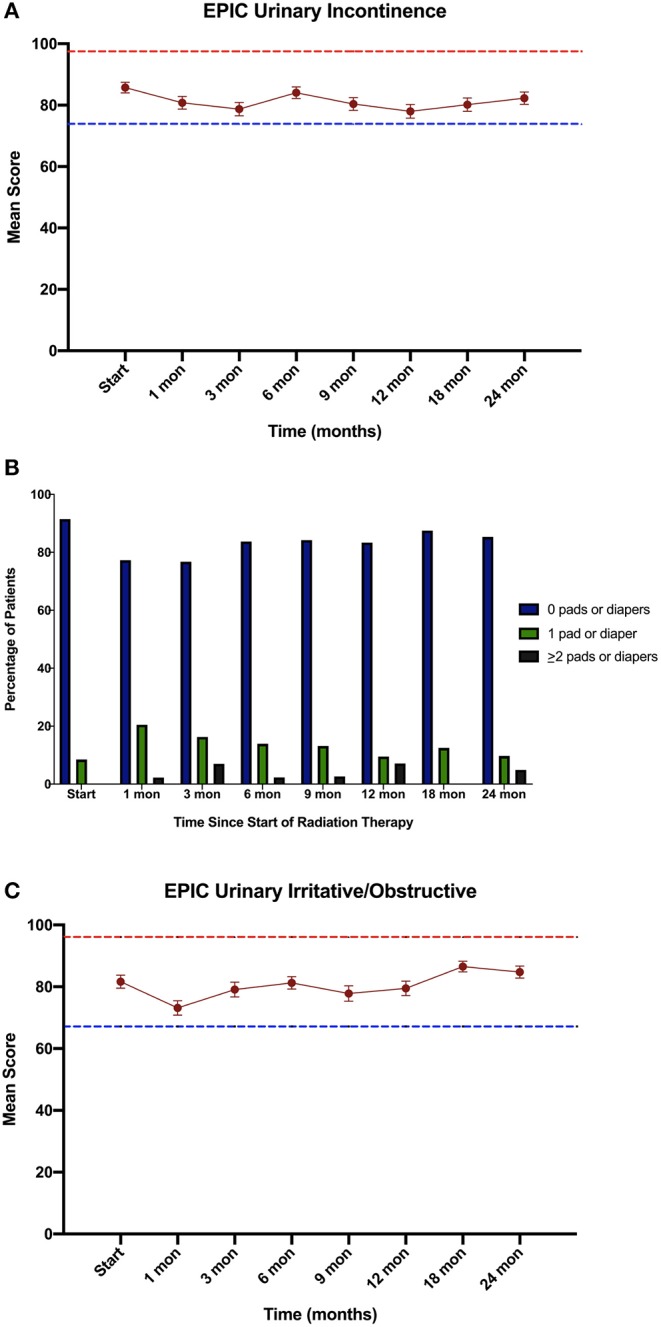
Urinary Quality of Life using the Expanded Prostate Cancer Index Composite (EPIC) for the **(A)** urinary incontinence, **(B)** Percentage of pad usage corresponding to EPIC question 27, and **(C)** urinary irritative/obstructive domains. EPIC scores range from 0 – 100 with higher values representing a more favorable health-related QOL. Error bars indicate SEM. Dashed lines represent the calculated minimally important difference values.

Consistent with their history of BPH treatment, the majority of patients had lower urinary tract symptoms prior to treatment (24) and decreased baseline urinary function (14). Baseline quality of life scores are shown in Table 4. Approximately 57 percent of patients had moderate to severe urinary tract symptoms prior to SBRT (Baseline IPSS >8) with a median baseline IPSS of 9. There was no clinically or statistically significant change in the IPSS score in the first 2 years following treatment (*p* = 0.125, MID 3.5) (Figure 3). The baseline urinary incontinence EPIC score was 85.7 (Table 4). It fell to 78.7 at 3 months (Figure 4A). By 24 months, the scores in the urinary incontinence domain increased to 82.3 back to baseline (*p* = 0.125, 95% CI −14.28 to −21.27). There was no clinically significant worsening of incontinence symptoms (MID 11.8). Likewise, there was no significant difference in pad usage over time (Figure 4B) (*X*^2^ = 5.473, *p* = 0.6025). Urinary irritative/obstructive symptoms fell from 81.6 at start of treatment (Table 4) to 73.2 at 1 month (Figure 4C). At 24 months, the EPIC irritative/obstructive score had increased back to 84.8 (*p* = 0.125, 95% CI −15.24 to −26.85). There was no clinically significant worsening of irritative/obstructive symptoms (MID 14.5).

**Table 4:** Baseline toxicity scores by IPSS and EPIC-26 urinary incontinence and irritative/obstructive domains.

**% Patients (*****n*** **=** **47)**			
**Baseline IPSS score**			
0–7 (mild)		42.6%	
8–19 (moderate)		48.9%	
>20 (severe)		8.5%	
	**Mean**	**SD**	**MID**
**Baseline EPIC-26 summary Score**			
Urinary Incontinence Domain	85.7	23.6	11.8
Urinary Irritative/obstructive Domain	81.6	28.9	14.5

## Discussion

Pre-treatment TURP has been shown to increase the risk of urinary toxicities following various radiation therapies including EBRT and brachytherapy (Table 5) (21, 25–28). The evidence is conflicting on whether TURP improves or worsens acute GU toxicity, but the impacts seem to have additive effects for late GU toxicity (29). This study aimed to assess the safety of performing prostate SBRT in patients with prior TURP. SBRT was generally well-tolerated in this population with rare high-grade acute toxicity (Table 2). Cumulative late ≥grade 2 and ≥grade 3 GU toxicity was observed in 49 and 6.4% of patients, respectively. It should be noted that the seemingly high rate of grade 2 GU toxicity was due to use of alpha-antagonists, 5 alpha reductase inhibitors and antimuscarinics for transient urinary symptoms (Figure 2). It is encouraging that only a few patients required fulguration or repeat TURP. Four patients were found to have urethral strictures/bladder neck contractures during cystoscopic evaluation for hematuria. However, in two of these patients, the strictures pre-dated SBRT treatment and were likely complications from their prior TURPs (9). Subclinical strictures with mild symptoms after TURP are common (9). Therefore, it is not possible to determine the cause of all strictures observed in post-SBRT cystoscopies. In this report we have included all strictures, regardless of presumed etiology.

**Table 5:** Summary of late grade 3 toxicities reported for various radiation therapy techniques in individuals who have undergone TURP.

**References**	**Institution/Trial**	**Technique**	**Dose (Gy)**	**Median follow-up (years)**	**Pts**	**Gr 3 GU (%)**
Devisetty et al. (25)	University of Chicago/Emory	3D-CRT + IMRT	70	3.3	71	7.0%
Lee et al. (26)	University of Florida	Proton	78	5.3	69	12.3%
Luo et al. (27)	Kaohsiung, Taiwan	HDR BT (3 fxn) + EBRT	12.6 + 37.8	4.2	32	3%
Demanes et al. (28)	California Endocurietherapy Cancer Center	HDR BT + EBRT	22–24 + 36	7.25	36	13.9%
Our population without TURP (21)	Georgetown University	SBRT	35–36.25	2.3	100	1%
Our study population	Georgetown University	SBRT	35–36.25	4.0	47	6.4%

Greater than 50% of our post-TURP patients experienced at least one episode of transient hematuria following SBRT. We have previously reported on hematuria rates in our total prostate cancer population undergoing SBRT. In that population, 18.3% of patients reported at least one episode of hematuria following SBRT with median time to hematuria being 13.8 months (29). In this study we show that 51.1% of our post-TURP patient reported at least one episode of hematuria following SBRT with median time to hematuria at 10.5 months. Of those, 81.8% resolved within the next timeline. In the majority of patients, hematuria resolved on its own with only six patients requiring medical management.

Patients in this study had poor baseline urinary quality of life with the majority had moderate urinary symptoms (IPSS >9). In prostate cancer patients treated with radiation, baseline EPIC urinary scores are commonly 90 (30–32). Our Post-TURP baseline urinary EPIC irritative and incontinence scores were in the low of 80 s. Lee et al. reported on patients with prior TURP undergoing proton therapy for their prostate cancer (26). Likewise, they found that pretreatment TURP was associated with inferior physician reported toxicity and patient-reported QOL. It is reassuring that in this study the post-TURP's patients' urinary quality of life did not significantly decline in the first 2 years following SBRT (33).

The mechanism for increased late morbidity following SBRT after TURP is unclear. It has been proposed that devascularization of the urethra following TURP lessens the ability of the mucosa to repair DNA damage (34). In addition, TURP defects expand the size of the prostatic urethra increasing the amount of mucosa exposed to high radiation doses. Finally, post-TURP patients are typically elderly with a high level of comorbidity prior to treatment, which may increase their baseline risk of late GU toxicity (35).

Limiting the radiation dose to the TURP-defect could reduce the risk of hematuria. However, urethral sparing with IMRT increases the risk of local recurrence (36) and may increase urinary morbidity by increasing the radiation dose to the bladder neck. Thus, we have modified our institutional protocol with the aim of reducing urinary symptoms without increasing the risk of local recurrence by employing urethral dose reduction (32). This approach has previously been applied effectively with brachytherapy (37). Specifically, we now contour the TURP cavity on the treatment planning scans and limit the point dose to 42 Gy. We hope this will reduce the rate of high-grade urinary toxicity in the future. Longer term follow up will be required to determine periurethral recurrence rae and overall toxicity in this population.

## Conclusions

In patients with prior TURP, prostate SBRT was well-tolerated. GU toxicity rates were comparable to similar patients treated with conventionally fractionated radiation therapy or brachytherapy. Urinary quality of life was poor at baseline, but did not worsen clinically over time. Stricter dosimetric criteria could potentially improve the rate of high-grade late toxicity, but may increase the risk of peri-urethral recurrence.

## Data Availability Statement

The raw data supporting the conclusions of this article will be made available by the authors, without undue reservation, to any qualified researcher.

## Ethics Statement

The studies involving human participants were reviewed and approved by The Georgetown University Institutional Review Board (IRB#: 2009-510). The patients/participants provided their written informed consent to participate in this study.

## Author Contributions

AP and NA were the lead authors, who participated in data collection, data analysis, manuscript drafting, table/figure creation, and manuscript revision. SSh aided in data collection. SK, HT, and SD participated in manuscript drafting and data analysis. TY and MA participated in clinical data collection. MD contributed to the study design and clinical data collection. SL developed the majority of patients' SBRT treatment plans and contributed to the data analysis and interpretation. BC and MG are senior authors who aided in reviewing the manuscript. PK aided in manuscript review and figure creation. RH and JL participated in manuscript review. SC was the principal investigator who initially developed the concept of the study and the design, aided in data collection, and drafted and revised the manuscript. SSu is a senior author who organized the data and participated in its analysis. All authors contributed to manuscript revision, read, and approved the submitted version.

## Conflict of Interest

SC and BC serve as a clinical consultant to Accuray Inc. The remaining authors declare that the research was conducted in the absence of any commercial or financial relationships that could be construed as a potential conflict of interest.

## Abbreviations

ADT: androgen deprivation therapy
BPH: Benign Prostatic Hyperplasia
CCI: Charlson Comorbidity Index
CT: computerized tomography
CTC: Common Toxicity Criteria
CTV: clinical target volume
DVH: dose-volume histogram
EBRT: external beam radiation therapy
EPIC: expanded prostate cancer index composite
GU: genitourinary
IMRT: intensity modulated radiation therapy
IPSS: international prostate symptom score
MRI: magnetic resonance imaging
PTV: planning target volume
QoL: quality of life
SBRT: stereotactic body radiation therapy
TURP: transurethral resection of the prostate.
